# Preliminary evaluation of the psychometric properties of the Italian version of the Psychotic Symptom Rating Scales (PSYRATS)

**DOI:** 10.1192/j.eurpsy.2025.2212

**Published:** 2025-08-26

**Authors:** S. Costantini, A. Di Lisi, L. Ghidetti, F. Cappella, L. Orlando, F. Catalano, M. Benassi, R. P. Sant’Angelo

**Affiliations:** 1 Mental Health, Ausl Romagna; 2 Psychology, University of Bologna, Cesena; 3 Psychiatry, University of Bologna, Bologna, Italy; 4 Psychiatry, South London and Maudsley NHS Foundation Trust, London, United Kingdom; 5 Mental Health Department, Ausl Romagna, Cesena, Italy

## Abstract

**Introduction:**

Most of the validated Italian scales for assessing psychotic symptoms don’t analyze specific symptoms like delusions and hallucinations in detail, but rather measure a wide range of experiences and behaviors in a general way. Scales such as PANSS, SAPS, and Sistema3 include only a few items on delusions and hallucinations, without considering the multidimensional characteristics of these symptoms, such as the degree of conviction, distress, duration, and perceived reality of the hallucinations. The Psychotic Symptom Rating Scales (PSYRATS) is the most widely used scale globally for measuring the severity of hallucinations and delusions through a semi-structured interview, but it has never been translated into Italian.

**Objectives:**

The aim of this study is to conduct a preliminary evaluation of the psychometric properties of the Italian version of the PSYRATs.

**Methods:**

The participants were recruited from among adult patients who were able to communicate fluently in Italian and were admitted to the Psychiatry Department of the Cesena Hospital. Eligibility depended on the presence of persistent delusions or auditory hallucinations in the week prior.

The hospitalized patients were assessed using the Italian version of the PSYRATS, the Positive And Negative Syndrome Scale (PANSS), and the Brief Psychiatric Rating Scale (BPRS). The reliability of the scales was assessed using Cronbach’s Alpha, and the relationship between the variables was analyzed using Spearman’s correlation.

**Results:**

The sample consisted of 25 participants (5 female, 20 male), with a mean age of 43 years and an average educational level of 11 years. A good internal consistency was observed for the “Auditory Hallucinations” scale (Alpha = .92) and for the “Delusions” scale (Alpha = .97). Spearman’s correlation analysis revealed that the “Total PSYRATs” scale has strong correlations with the “Auditory Hallucinations” scale (Rho = .93; p = .001) and the “Delusions” scale (Rho = .45; p = .02). Valid correlation indices were also noted between the “Total PSYRATs” and “Total BPRS” scales (Rho = .62; p = .001) and the PANSS scale (Rho = .45; p = .02).

**Image 1:**

**Figure:**
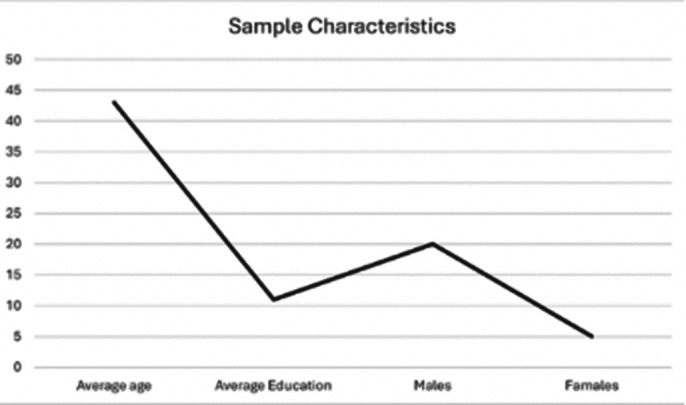

**Image 2:**

**Figure:**
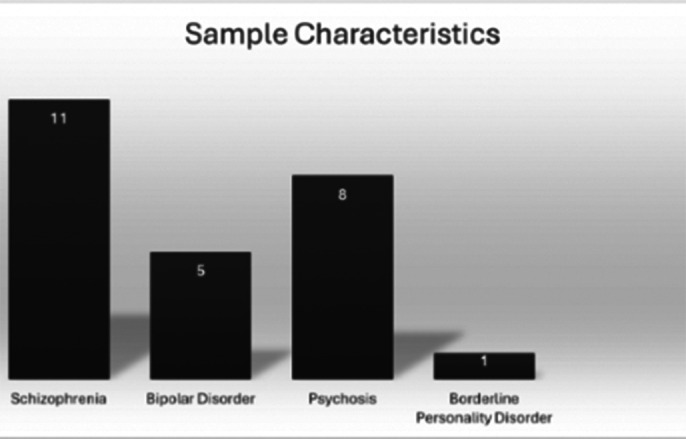

**Image 3:**

**Figure:**
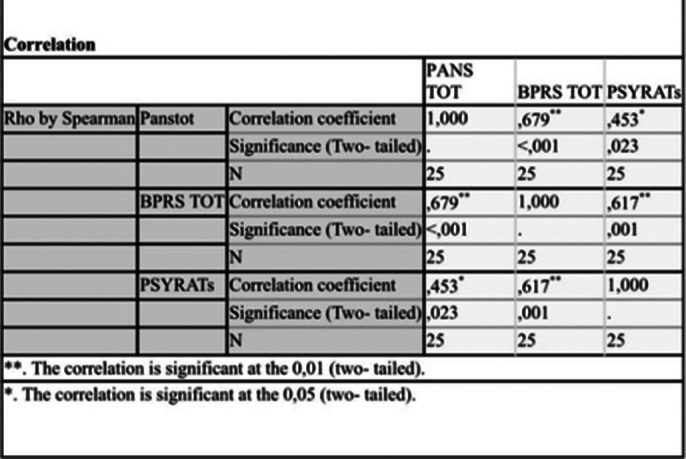

**Conclusions:**

The preliminary data demonstrate good internal consistency and convergent validity of the Italian version of the PSYRATS; therefore, this instrument appears to be a valid measure for the assessment of hallucinations and delusions. The results of this study are limited by the relatively small sample size, making it essential to repeat the analyses on a larger population.

**Disclosure of Interest:**

None Declared

